# Mechanical force regulates Sox9 expression at the developing enthesis

**DOI:** 10.1242/dev.201141

**Published:** 2023-08-18

**Authors:** Arul Subramanian, Lauren F. Kanzaki, Thomas F. Schilling

**Affiliations:** Department of Developmental and Cell Biology, University of California, Irvine, CA 92697, USA

**Keywords:** Mechanotransduction, Tendon, Tenocyte differentiation, Enthesis, Zebrafish

## Abstract

Entheses transmit force from tendons and ligaments to the skeleton. Regional organization of enthesis extracellular matrix (ECM) generates differences in stiffness required for force transmission. Two key transcription factors co-expressed in entheseal tenocytes, scleraxis (Scx) and Sox9, directly control production of enthesis ECM components. Formation of embryonic craniofacial entheses in zebrafish coincides with onset of jaw movements, possibly in response to the force of muscle contraction. We show dynamic changes in *scxa* and *sox9a* mRNA levels in subsets of entheseal tenocytes that correlate with their roles in force transmission. We also show that transcription of a direct target of Scxa, Col1a, in enthesis ECM is regulated by the ratio of *scxa* to *sox9a* expression. Eliminating muscle contraction by paralyzing embryos during early stages of musculoskeletal differentiation alters relative levels of *scxa* and *sox9a* in entheses, primarily owing to increased *sox9a* expression. Force-dependent TGF-β (TGFβ) signaling is required to maintain this balance of *scxa* and *sox9a* expression. Thus, force from muscle contraction helps establish a balance of transcription factor expression that controls specialized ECM organization at the tendon enthesis and its ability to transmit force.

## Teaser

**Summary:** The role of mechanical force in regulating gene expression at the tendon-bone interface and the consequences for the structure of the tendon matrix.

## INTRODUCTION

Mechanical force plays crucial roles in development and maintenance of the musculoskeletal system. Connective tissues such as tendons transmit the forces of muscle contraction to skeletal elements. Effective transmission requires transferring a range of physical forces from soft muscle tissue to much stiffer cartilage or bone (Shaw and Benjamin, 2007; Thomopoulos et al., 2010; Zelzer et al., 2014). Specialized areas at the tendon-bone interface called entheses accommodate these physical constraints using a unique fibrocartilaginous extracellular matrix (ECM) that varies in composition along the length of the enthesis (Cury et al., 2016; Rufai et al., 1995; Apostolakos et al., 2014). Osteo-tendinous junction (OTJ) entheses are often sites of tendon-associated injuries such as avulsion fractures, rotator cuff tears and chronic age-related diseases called entheseopathies, which are characterized by increased calcification (Shaw and Benjamin, 2007; Tadros et al., 2018). Treatments of either acute or chronic enthesis injuries are challenging because the ECM is never fully restored after healing. Thus, it is important to understand the mechanisms by which entheses form during development and respond to force to acquire their strength.

Entheses are associated with many different types of tendon and ligament attachments to bone and vary in ECM composition and organization depending on the force associated with the attachment (Benjamin et al., 2002; Benjamin and Ralphs, 1998; Hems and Tillmann, 2000; Apostolakos et al., 2014). Two crucial transcription factors specify cells and regulate expression of ECM components in a developing enthesis: the tenogenic fate determinant scleraxis, Scx, and the chondrogenic fate determinant Sox9 (Blitz et al., 2013; Sugimoto et al., 2013). Expression of Scx in mesenchymal stem cells (MSCs) drives them toward a tendon fibroblast (tenocyte) fate, whereas expression of Sox9 promotes cartilage differentiation (Akiyama et al., 2002, 2005). Co-expression of Scx and Sox9 has been described in cells of the OTJ enthesis of the Achilles tendon with the calcaneus as well as tendons attached to the mandible and supraspinatus-humerus (Blitz et al., 2013; Ideo et al., 2020; Roberts et al., 2019; Sugimoto et al., 2013; Kult et al., 2021). This is thought to be important for the establishment of the specialized ECM structure of these entheses, as both Scx and Sox9 directly regulate transcription of several collagens and other ECM components (Subramanian and Schilling, 2015). As a result, embryonic entheses have been defined by the co-expression of Scx and Sox9. In mice, *Scx* and *Sox9* exhibit graded expression at fibrocartilaginous enthesis attachment sites, with cells closer to the bone expressing higher levels of *Sox9* and cells closer to the tendon midbody and muscle expressing higher levels of *Scx* (Blitz et al., 2013; Sugimoto et al., 2013).This graded expression correlates with the gradient in stiffness and elasticity of enthesis tissue from bone to tendon, suggesting that it may play a causal role in tuning tendon ECM composition (Thomopoulos et al., 2010).

We have previously shown that mechanical force from muscle contraction controls transcriptional programs in tenocytes and the organization of tendon ECM in developing zebrafish (Subramanian et al., 2018). Loss of muscle in *Myod1^−/−^;Myf5^−/−^* mutant mice as well as induced paralysis in chick or mouse embryos disrupts the growth of bones and formation of bony eminences associated with entheses (Hall and Herring, 1990; Hosseini and Hogg, 1991; Rot-Nikcevic et al., 2006). For example, injection of alpha-Bungarotoxin (aBgtx) protein into the neonatal mouse brachial plexus to reversibly paralyze muscles disrupts collagen (Col) composition and other aspects of enthesis ECM organization, thereby compromising its function (Schwartz et al., 2013). However, it is not known how mechanical force regulates transcriptional dynamics during tenocyte differentiation at an enthesis or how this in turn affects interactions between tenocytes and the enthesis ECM.

Here, we describe the development of embryonic entheses during craniofacial development in zebrafish, and show that cells associated with muscle attachment sites on developing cartilages co-express *scxa* and *sox9a* transcripts, consistent with previously described presumptive enthesis progenitor identity (Sugimoto et al., 2013; Kult et al., 2021). We also describe the ECM organization at these and other developing cranial entheses, including the sternohyoideus enthesis, which elongates upon the onset of muscle contraction to resemble commonly studied entheses in long bones of mammals. Using both irreversible and reversible methods of paralysis, as well as a quantitative *in situ* hybridization method for measuring transcript levels, we show that mechanical force from muscle contraction regulates the establishment of graded *scxa* and *sox9a* expression at developing entheses (Subramanian et al., 2018; Niu et al., 2020). This is, in part, through TGFβ signaling, as pharmacological inhibition of TGFβ signaling also disrupts the ratio of *scxa*/*sox9a* expression along these developing entheses, suggesting that it plays an important mechanotransductive role in regulating the initial formation of entheses during embryonic musculoskeletal development.

## RESULTS

### *scxa* and *sox9a* are co-expressed at embryonic cranial cartilage-tendon attachments

**Fig. 1.**
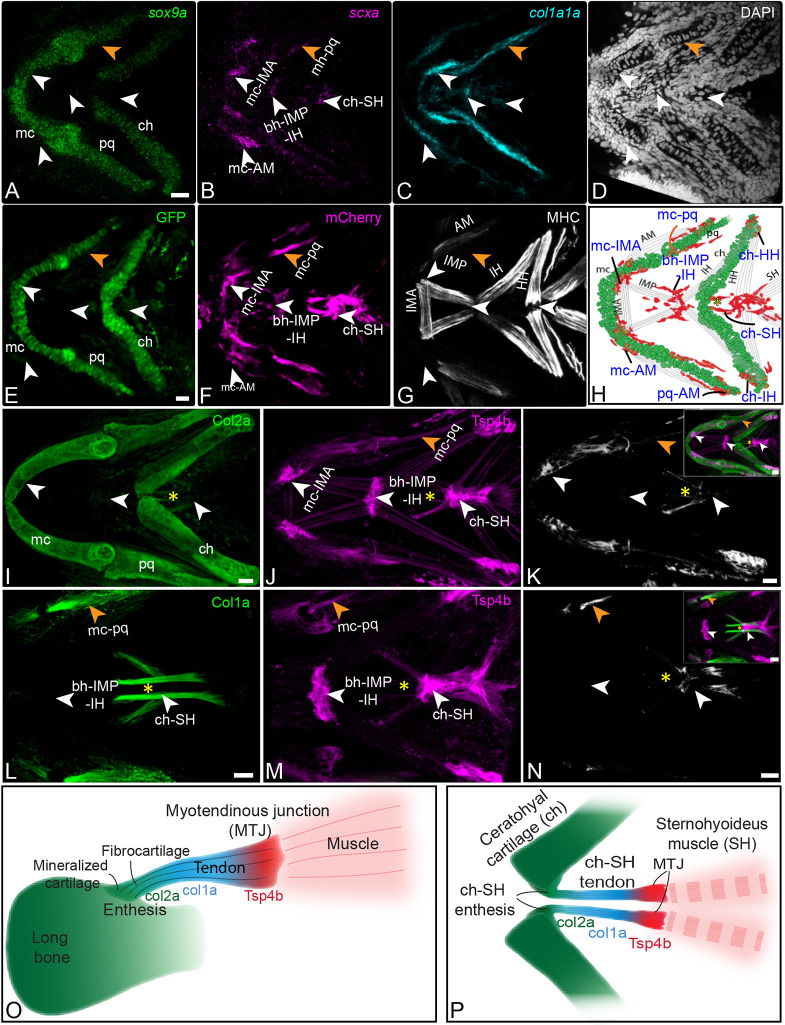
**Tenocytes at cranial muscle attachments express *scxa*, *sox9a* and *col1a*.** (A-C) Single-plane ventral views, anterior to the left, of 72 hpf zebrafish embryos showing *in situ* hybridization chain reaction (isHCR) staining for expression of *sox9a* (green), *scxa* (magenta) and *col1a1a* (cyan) mRNA at cranial muscle-cartilage attachments (entheses). (D) Similar view of cell nuclei labeled with DAPI. (E-G) *Z*-stack ventral views of 72 hpf Tg(*sox10:gfp;BAC scx:mCherry*) embryos stained by immunofluorescence for GFP (E), mCherry (F) and myosin heavy chain (MHC; G). (H) Cartoon showing a ventral view of a 72 hpf embryo illustrating chondrocytes expressing *sox9a* (green) and tenocytes expressing *scxa* (red), with dual expression of both genes in tenocytes of entheses. (I-K) *Z*-stack ventral views, anterior to the left, of 96 hpf embryos stained by immunofluorescence for Col2a (I), Tsp4b (J) and Col2a/Tsp4b colocalization (K). (L-N) *Z*-stack ventral views of 5 dpf embryos stained by immunofluorescence for Col1a (L), Tsp4b (M) and Col1a/Tsp4b colocalization (N). Arrowheads indicate tendons (white) and ligaments (orange) attaching with mc, ch, pq and bh cartilages. Yellow asterisks show the developing entheseal ECM of the ch-SH tendon. (O) Cartoon showing ECM organization of a tendon attaching a muscle to a mammalian long bone, with fibrocartilage and Col2a^+^ matrix near the bony junction (green), Col1a-rich matrix in the tendon midbody (blue) and Tsp4 (red) in the myotendinous junction (MTJ). (P) Cartoon showing ECM organization of the elongated tendon in zebrafish attaching the SH muscle to the ch cartilage, with similar Col2a^+^ (green), Col1a^+^ (blue) and Tsp4^+^ (red) defining similar regions. AM – adductor mandibularis; bh-IMP-IH, basihyal-intermandibularis posterior-interhyal; ch, ceratohyal; ch-HH, ceratohyal-hyohyal tendon; ch-IH, ceratohyal-interhyal tendon; ch-SH, ceratohyal-sternohyoideus tendon; HH, hyohyal; IMA; intermandibularis anterior; IMP, intermandibularis posterior; Mc, Meckels cartilage; mc-AM, Meckels adductor mandibularis tendon; mc-IMA, Meckels-intermandibularis anterior tendon; mc-pq, Meckels-palatoquadrate ligament; pq, palatoquadrate; pq-AM, palatoquadrate-adductor mandibularis tendon. Scale bars: 20 μm.

Early cartilages of the craniofacial skeleton and associated tendons/ligaments in zebrafish embryos differentiate between 48 and 72 hours postfertilization (hpf) coinciding with the onset of head and jaw movements (Schilling and Kimmel, 1997; Subramanian et al., 2018). We used *in situ* hybridization chain reaction (isHCR) to assess spatial patterns of *sox9a* and *scxa* expression at developing cranial muscle attachments and sites of co-expression (presumptive entheses) (Fig. 1). We performed three-color isHCR using sets of probes to detect the cartilage marker *sox9a* (Fig. 1A), and the tenocyte markers *scxa* (Fig. 1B) and *col1a1a* (Fig. 1C) in 72 hpf wild-type (WT) embryos. These were compared with 72 hpf WT Tg(BAC *scx:mCherry^fb301^; sox10:gfp^ir937^*) double transgenic embryos stained with antibodies against GFP to detect *sox10* in cartilage cells (Fig. 1E), mCherry to detect *scxa* in tenocytes (Fig. 1F) and myosin heavy chain (MHC) to mark jaw muscle fibers (Fig. 1G). In the ventral cartilages and muscles of the first and second pharyngeal arches, *sox9a* and *scxa* expression coincided with the GFP (chondrocytes) and mCherry (tenocytes) antibody signals, validating the isHCR probe set specificity.

In addition, we identified overlapping regions of expression of *scxa* and *sox9a* at many tendon-cartilage attachment sites that we hypothesized to be developing entheses (Fig. 1H). To aid in detecting enthesis cells and their 3D organization, we used DAPI to stain nuclei. By 72 hpf cranial tendons are at least partially functional and developing *scxa^+^* tenocytes have largely migrated to sites of tendon attachments to cartilage (Fig. 1H). To aid in visualizing ECM organization at entheses, we also stained 4 day postfertilization (dpf) and 5 dpf embryos with antibodies against Col2a to mark cartilage/fibrocartilage (Fig. 1I,K), Col1a to mark the tendon midbody (Fig. 1L,N) and Tsp4b to mark myotendinous junctions (MTJ) (J,K,M,N). We could not perform dual staining for Col2a and Col1a as their respective antibodies were raised in the same host species. In elongated tendons, such as those that connect the sternohyoideus (SH) muscle to the ceratohyal (ch) cartilage – hereafter referred to as the ch-SH attachment – Col2a labeling extended from ch posteriorly toward SH (Fig. 1I-K, yellow asterisk). This coincided with localization of Col1a in ch-SH (Fig. 1L-N, yellow asterisk), expression of which partially overlapped posteriorly with Tsp4b (Fig. 1K,N, insets). This colocalization of Col2a and Col1a at the anterior end of ch-SH is similar to fibrocartilage described in tendon insertions and entheses in mice and humans (Fig. 1O,P) (Thomopoulos et al., 2010; Apostolakos et al., 2014).

**Fig. 2.**
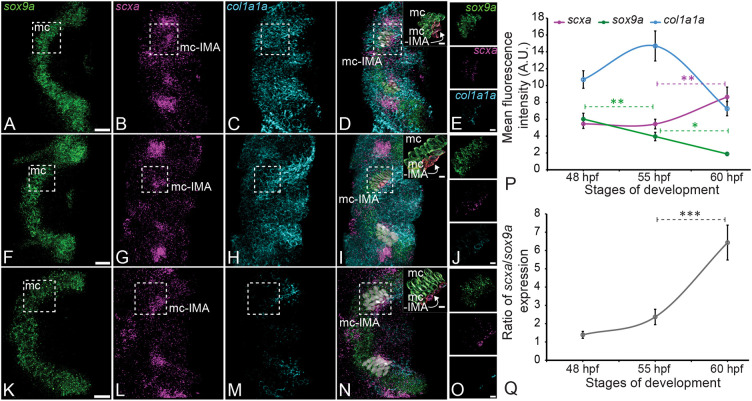
***scxa* and *sox9a* are co-expressed in tenocytes of putative entheses of cranial tendons during embryonic development.** (A-O) Ventral views, anterior to the left, of the developing mc cartilages of the lower jaws of zebrafish embryos showing isHCR staining for expression of *sox9a* (green), *scxa* (magenta) and *col1a1a* (cyan) mRNA at the mc-IMA enthesis at 48 hpf (A-E), 55 hpf (F-J) and 60 hpf (K-O). Surface volumes traced from DAPI-stained nuclei of cartilage (green) and tenocytes (red) within the enthesis region (dashed boxes) were created to quantify expression (D,E,I,J,N,O). (P,Q) Plots show quantification of fluorescence intensity within the tenocyte surface volume for *scxa* and *sox9a* expression and mean fluorescence level (P), and ratios of *scxa* to *sox9a* for the stages indicated (Q). *n*=4 embryos per stage, eight cells per embryo. Error bars show standard deviation. Linear mixed effects model was created and Tukey post-hoc pairwise comparison was performed. *P<0.05, **P<0.01, ***P<0.001. Scale bars: 20 μm (A-D,F-I,K-N); 5 μm (insets, E,J,O).

As isHCR signal amplification depends on enzyme-free isothermal hybridization of labeled amplifiers with linkers on the probes, the fluorescent signal remains restricted to the vicinity of the expressed mRNA molecules, allowing a semi-quantitative estimate of expression levels (Choi et al., 2016). We focused on the overlapping regions of expression of *scxa* and *sox9a* in putative entheseal tenocytes at the ch-interhyal (IH) muscle attachment, hereafter referred to as ch-IH tendon (Fig. 1H), and Meckel's (mc)-intermadibularis anterior (IMA) muscle, hereafter referred to as mc-IMA, as these attachments initially form between 48 and 72 hpf and become functional in jaw movements during this period. We performed isHCR hybridization for *sox9a* and *scxa* in WT embryos at 48, 55 and 60 hpf, then quantified expression in tenocytes as defined by expression of *scxa* at the mc-IMA attachment by measuring mean total tenocyte fluorescence (Fig. 2A-O) in 3D regions of interest (ROIs) created per cell using the DAPI nuclear signal. Developing cranial tendons are populated by ∼30-50 *scxa*-expressing cells and only a small fraction of these cells lies at the interface of the tendon and cartilage – the future enthesis. After examining the tendon-cartilage junction in a *z*-stack, we selected eight cells per embryo (∼four cells for each attachment) to be quantified in five embryos, which was ∼70% of the tenocytes at the enthesis that co-express *scxa* and *sox9a*. Our analysis showed a decrease in *sox9a* expression and an increase in *col1a1a* expression between 48 and 55 hpf, followed by a decrease in *sox9a* expression between 55 and 60 hpf and increase in *scxa* expression from 48-60 hpf (Fig. 2P), As a result, the ratio of *scxa* versus *sox9a* increased, particularly from 55 to 60 hpf (Fig. 2Q). To determine ratios of *scxa* and *sox9a* in tenocytes based on their relative positions within putative entheses (i.e. distance from the cartilage attachment), we measured *scxa* and *sox9a* expression at positions 1-4, with ‘1’ being a cell located closest to the cartilage and ‘4’ a cell further from the cartilage, at 48, 55, 60 and 72 hpf in the mc-IMA enthesis (Fig. S1A-K). We found no significant positional variation in *scxa* expression except for a decrease at 72 hpf between cells in positions 2 and 3 (Fig. S1C). In contrast, *sox9a* expression was highest in tenocytes closest to the OTJ (positions 1 and 2) from 48 to 55 hpf, with a gradual decrease progressively further away. (Fig. S1F). At 72 hpf *sox9a* expression was low in cells at all positions, with lowest levels at position 4. These dynamics are reflected in the much higher ratio of *scxa* versus *sox9a* expression in cells further from the cartilage attachment. (Fig. S1L). Correspondingly we observed higher expression of *col1a1a* at 60 hpf and 72 hpf in tenocytes adjacent to the cartilage, with steep decreases in expression at positions 3 and 4. These results suggest that the differential expression of *scxa* and *sox9a* seen at mature entheses arises at embryonic stages of cartilage and muscle differentiation and correlates with cell position within an enthesis primordium.

### Paralysis alters the ratio of *scxa* and *sox9* expression at entheses through regulation of *sox9a* expression

**Fig. 3.**
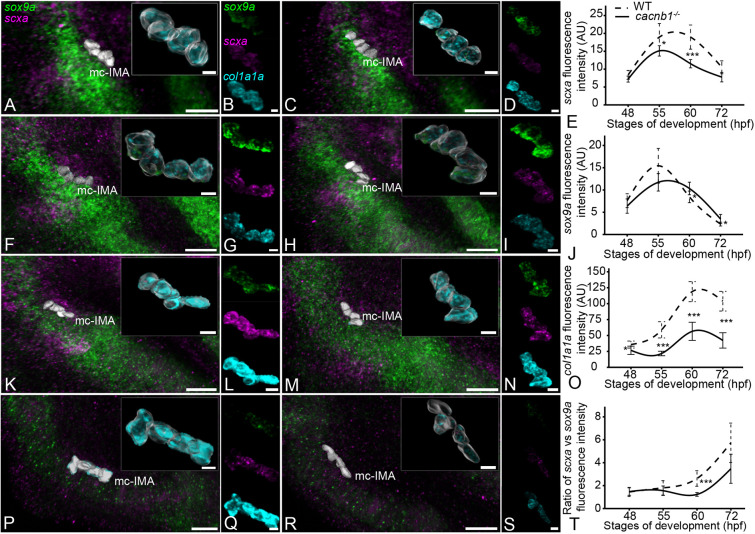
**Paralysis regulates expression of *scxa*, *sox9a* and *col1a1a* in the embryonic mc-IMA enthesis.** (A-S) Ventral views, anterior to the left, of wild-type (WT) (A,B,F,G,K,L,P,Q) and *cacnb1^−/−^* (C,D,H,I,M,N,R,S) embryos showing expression of *scxa* (magenta), *sox9a* (green) and *col1a1a* (cyan) genes at the mc-IMA tendon enthesis at 48 hpf (A-D), 55 hpf (F-I), 60 hpf (K-N) and 72 hpf (P-S). Plots show quantification of *scxa* (E), *sox9a* (J), *col1a1a* (O) expression and ratio of *scxa* versus *sox9a* expression (T) in WT (dotted line) and *cacnb1^−/−^* (solid line) tenocytes for the stages indicated. Error bars show standard deviation. Linear mixed effects model was created and Tukey post-hoc pairwise comparison was performed. *P<0.05, ***P<0.001. Scale bars: 20 μm (A,C,F,H,K,M,P,R); 5 μm (insets, B,D,G,I,L,N,Q,S).

The temporal correlation between the differential expression of *scxa* and *sox9a* and the onset of coordinated jaw muscle contraction (60-72 hpf) suggests that force from muscle contraction regulates these expression levels at embryonic entheses. To test this hypothesis, we quantified expression in tenocytes of the mc-IMA in paralyzed embryos. Homozygous mutants lacking the function of a *calcium channel, voltage-dependent, beta 1 subunit* (*cacnb1^−/−^*), required for myofiber contraction, show no movement as embryos but develop with no obvious morphological differences from WT siblings for the first 4 dpf (Subramanian et al., 2018). We quantified expression of *scxa* and *sox9a* in tenocytes at mc-IMA attachments in *cacnb1^−/−^* embryos at 48, 55, 60 and 72 hpf and changes in expression levels from WT for *scxa* (decreases of 39% at 60 hpf and 26% at 72 hpf), *sox9a* (increases of 1.2-fold at 60 hpf and 1.5-fold at 72 hpf) and *col1a1a* (decreases of 52% at 60 hpf and 59% at 72 hpf) (Fig. 3A-O; Fig. S2A-C). A lack of the active jaw movements in *cacnb1^−/−^* embryos correlated with a failure to upregulate *scxa* and downregulate *sox9a* expression to the same extent seen in WT in tenocytes of the mc-IMA attachment, resulting in a 53% and 40% reduction in the ratio of *scxa* to *sox9a* at 60 and 72 hpf, respectively (Fig. 3T; Fig. S2D). We saw no difference in this ratio at 48 hpf, before any cranial muscle movements, but *cacnb1^−/−^* embryos showed a slightly reduced ratio of *scxa* to *sox9a* starting at 55 hpf, before there were coordinated cranial muscle contractions but when individual muscle fibers still exhibited random sporadic contractions.

To determine whether muscle contraction has similar effects on gene expression in other tendons besides mc-IMA and their developing entheses, we quantified expression of *scxa*, *sox9a* and *col1a1a* in tenocytes along the length of the ch-SH tendon (see Fig. 1). Cells at positions 1-5, which are closest to the ch cartilage, showed strong changes in expression of *sox9a*, *scxa* and *col1a1a* similar to our observations in the mc-IMA enthesis. *sox9a* expression was higher in cells in position 1 in *cacnb1^−/−^* embryos compared with cells at position 1 in WT controls. *sox9a* levels gradually decreased from anterior to posterior (1-5) along the tendon, which correlated with a decrease in the ratio of *scxa* to *sox9a* at the tenocytes closer to the enthesis in *cacnb1^−/−^* embryos (Fig. S3A-J). These effects of muscle activity, although stronger in tenocytes at the enthesis, were also noticed in chondrocytes, as we observed a modest change in s*ox9a* expression in adjacent chondrocytes in *cacnb1^−/−^* embryos when compared with WT (Fig. S4A-E).

**Fig. 4.**
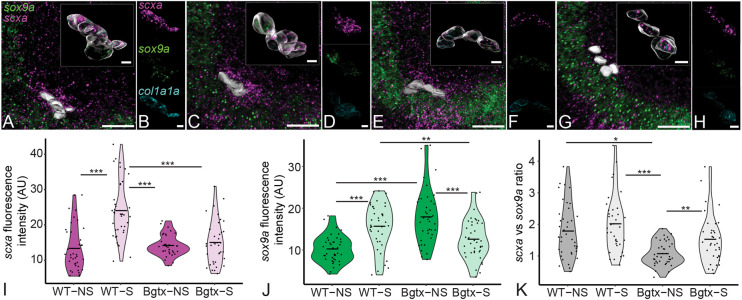
**Muscle contraction regulates expression of *scxa*, *sox9a* and *col1a1a* in the embryonic enthesis.** (A-H) Ventral views, anterior to the left, of wild-type not stimulated (WT-NS) (A,B), wild-type stimulated (WT-S) (C,D), *aBgtx*-injected not stimulated (Bgtx-NS) and *aBgtx*-injected stimulated (Bgtx-S) embryos showing expression of *scxa* (magenta), *sox9a* (green) and *col1a1a* (cyan) genes at the mc-IMA enthesis at 60 hpf. (I,J) Violin plots showing expression level as quantified by measuring fluorescence intensity from isHCRs of *scxa* (I) and *sox9a* (J) in WT-NS and Bgtx-NS and WT-S and Bgtx-S at 72 hpf. (K) Violin plots showing ratios of *scxa* versus *sox9a* expression in in WT-NS and Bgtx-NS and WT-S and Bgtx-S at 72 hpf. *N*=4 embryos per developmental stage and nine tenocytes per embryo. Horizontal line in each distribution shows median value. Linear mixed effects model was created and Tukey post-hoc pairwise comparison was performed. *P<0.05, **P<0.01, ***P<0.001. Scale bars: 20 μm (A,C,E,G); 5 μm (insets, B,D,F,H).

To confirm that the loss of muscle contraction and not some other role for Cacnb1 was responsible for these changes in *scxa* and *sox9a* expression, we injected *aBgtx* RNA into one-cell-stage embryos to block acetyl choline receptors in neuromuscular synapses and result in complete paralysis of zebrafish embryos for 3-4 days without noticeably disrupting other aspects of development (Subramanian et al., 2018). Similar to our results in *cacnb1^−/−^* mutant embryos, the ratio of *scxa* to *sox9*a expression was significantly reduced (65%) in *aBgtx*-injected (*aBgtx*-inj) embryos at 72 hpf (Fig. 4A,B,E,F,K). These results further support that these changes in gene expression at developing entheses are due to mechanical force from muscle contraction.

### Restoring muscle contraction rescues *scxa* and *sox9*a expression at developing entheses following paralysis

If the hypothesis that force from muscle contraction drives the changes in gene expression holds true, then restoring muscle contraction in paralyzed embryos should rescue the relative expression levels of *scxa* and *sox9a* at the enthesis. To test this, we induced muscle contractions in *aBgtx*-inj paralyzed embryos at 72 hpf by electrical stimulation using a Grass simulator (20 V for 2 min) as described previously (Subramanian et al., 2018; Subramanian and Schilling, 2014). Quantification of *scxa* and *sox9a* expression using isHCR showed that, whereas stimulation upregulated expression of both genes in WT embryos (Fig. 4A-D,I,J), there was no significant change in *scxa* expression between *aBgtx*-inj and stimulated *aBgtx*-inj embryos (Fig. 4E-H,I). In contrast, *sox9a* expression decreased significantly in stimulated *aBgtx*-inj embryos when compared with non-stimulated *aBgtx*-inj animals (Fig. 4E-H,J). This resulted in a marked increase in the ratio of *scxa* to *sox9*a expression in stimulated *aBgtx*-inj embryos to levels similar to non-stimulated WT (Fig. 4K). These results suggest that mechanical force from muscle activity maintains a balance of *scxa* to *sox9a* expression at the developing enthesis primarily through repression of *sox9a* in tenocytes.

### TGFβ signaling regulates gene expression at developing entheses

Several studies, including ours, have shown that mechanical force regulates TGFβ signaling in the musculoskeletal system (Havis et al., 2016; Maeda et al., 2011; Subramanian et al., 2018; Liu et al., 2015). TGFβ signaling also regulates Scx expression and tendon development in mammalian limbs (Pryce et al., 2009; Tan et al., 2020). TGFβ signaling actively regulates tenocyte morphogenesis in the zebrafish trunk (Subramanian et al., 2018). To test whether TGFβ similarly controls transcriptional regulation in developing cranial tendons, we stained 60 hpf embryos with antibodies against phosphorylated SMAD3 (pSMAD3) and quantified expression of pSMAD3 in entheseal tenocytes. We observed significant reductions in pSMAD3 localization in the nuclei of the tenocytes at the developing enthesis (Fig. S5).

**Fig. 5.**
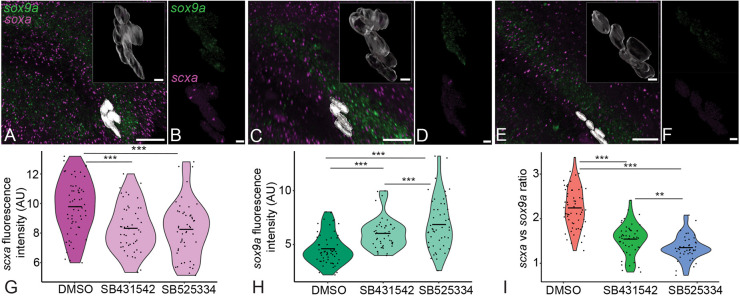
**Inhibition of TGFβ signaling regulates *scxa* and *sox9a* expression in embryonic entheseal tenocytes.** (A-F) Ventral views, anterior to the left, of 72 hpf embryos showing fluorescence signal from isHCRs of *scxa* and *sox9a* of ch-IH entheseal tenocytes treated with DMSO (A,B), 50 μM SB431542 (C,D) or 50μM SB525334 (E,F). (G,H) Violin plots depicting fluorescence intensity (AU) of *scxa* (G) and *sox9a* (H) in tenocytes treated with DMSO, 50 μM SB431542 or 50 μM SB525334. Each data point represents an individual tenocyte from four embryos (ten tenocytes per embryo) for each condition. (I) Violin plot depicting ratio of *scxa* to *sox9a* expression as fluorescence intensity. Horizontal line in each distribution shows median value. Linear mixed effects model was created and Tukey post-hoc pairwise comparison was performed. **P<0.01, ***P<0.001. Scale bars: 20 μm (A,C,E); 5 μm (insets, B,D,F).

As tools for tissue-specific ablation of TGFβ signaling are currently unavailable in zebrafish, to test whether the balance of *scxa* and *sox9a* expression that we observed at mc-IMA, ch-IH and ch-SH attachments of zebrafish requires TGFβ signaling, we used small molecule TGFβ signaling antagonists. We have previously shown that SB431542, a potent selective inhibitor of ALK5 receptors, effectively reduces TGFβ responses in tenocytes as assayed by immunostaining for pSMAD3 (Subramanian et al., 2018). We incubated embryos in either 50 µM SB431542 or another selective inhibitor of ALK5, SB525334 (similarly at 50 µM), at 60 hpf in DMSO containing embryo medium for 12 h. At 72 hpf, the embryos were fixed and divided into two batches. One batch was used to perform immunohistochemistry to assess the localization of pSMAD3. The other batch was used to perform isHCR and quantify and compare *scxa* and *sox9a* expression at the enthesis between DMSO-treated controls and inhibitor-treated embryos. Immunostaining of the embryos with anti-pSMAD3 antibody showed reduced nuclear pSMAD3 in tenocytes (SB431542-treated) at the mc-IMA attachment interface (∼15% lower than DMSO) with a stronger effect in SB525334-treated embryos (∼27% lower than DMSO) (Fig. S6). The remaining embryos used for isHCR showed significantly reduced *scxa* expression (∼14%) and a corresponding increase (∼40%) in *sox9a* expression in the tenocytes of both SB431542- and SB525334-treated embryos compared with the DMSO-treated control embryos, which leads to a lowered ratio (∼37% lower) of *scxa* to *sox9a* in the inhibitor-treated embryos (Fig. 5). These results, along with the reduction of pSMAD3 localization observed in paralyzed embryos, suggest that TGFβ signaling is required for maintaining the relative expression of *scxa* and *sox9a* at developing entheses.

## DISCUSSION

The classical view of an enthesis is a region of specialized ECM and cells (tenocytes, fibrocartilage) at the skeletal insertion site of a tendon or ligament that helps transfer mechanical load from soft to hard tissue. Cells of entheses in developing tetrapod limbs co-express *Scx* and *Sox9*, the balance of which has been hypothesized to regulate ECM composition along the length of a tendon in response to force (Felsenthal and Zelzer, 2017; Saito et al., 2021). How entheses first form in the embryo, their initial expression of *Scx* and *Sox9*, and their responses to mechanical force at the level of individual cells remain unclear. Here, we show that small groups of tenocytes co-express *scxa* and *sox9a* in craniofacial tendon attachments to cartilage in embryonic zebrafish that prefigure future entheses on later endochondral/perichondral bones. We find that tenocytes at these early entheses exhibit heterogeneity in levels of *sox9a* transcripts that correlate with their positions i.e. *sox9a* levels decrease further from the cartilage attachment. This transcriptional heterogeneity occurs in tendons with different types of attachments – including flattened sheet-like attachments such as mc-IMA and long extended tendons such as ch-SH. Similar to the ECM organization described in mouse and human entheses, zebrafish enthesis also show graded Col2a and Col1a expression, highest in fibrocartilage adjacent to skeletal attachments, and higher Tsp4b closer to the MTJ. We show that these position-specific differences in expression are amplified at the onset of active muscle contraction and jaw movements in embryos. We further show that mechanical force from muscle contraction regulates the *scxa*/*sox9a* ratio in individual tenocytes *in vivo*, primarily through repression of *sox9a*, which inversely correlates with *col1a1a* expression in the tenocytes at the entheseal and tendon ECM and helps maintain this ratio downstream of force to promote enthesis development.

**Fig. 6.**
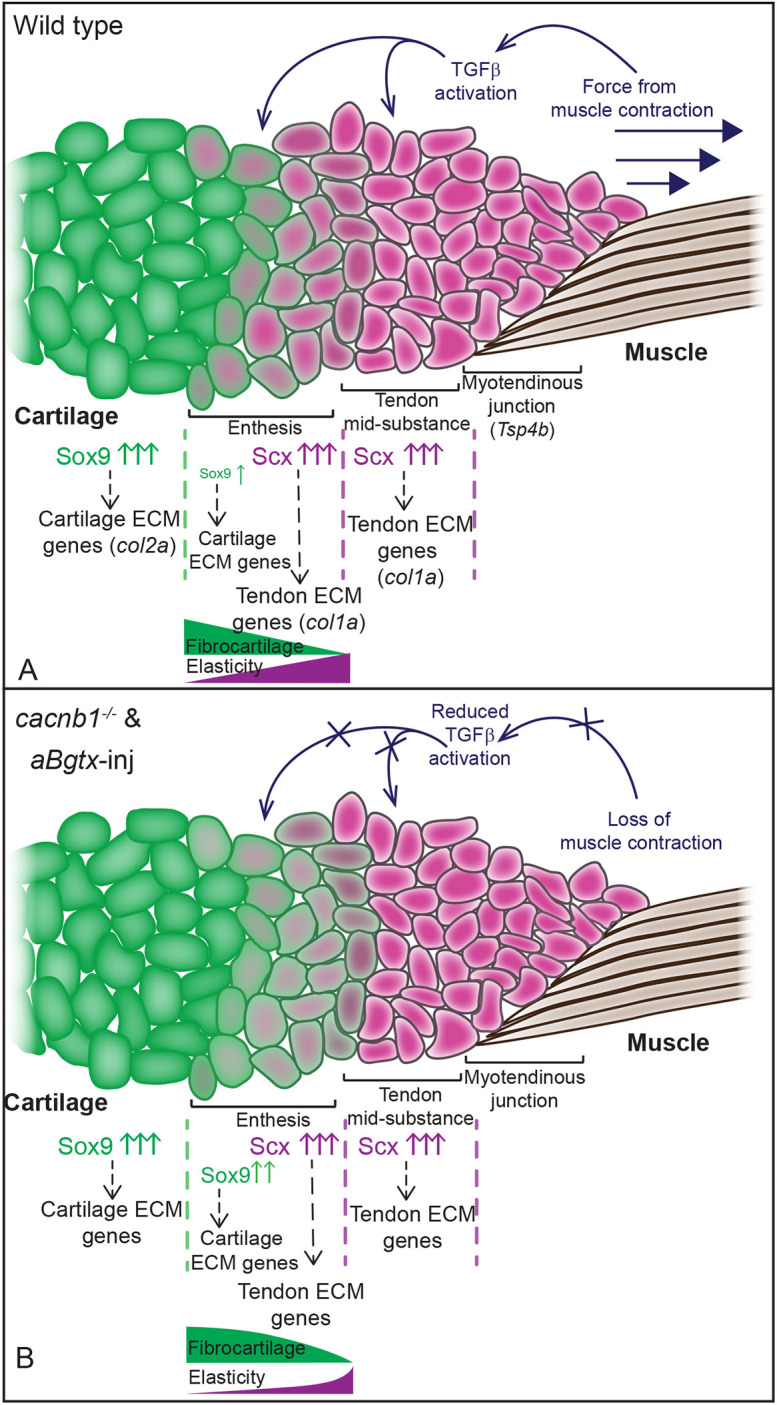
**Model for role of muscle contraction in enthesis development.** (A) Mechanical force from muscle contraction regulates the balance of *sox9a* and *scxa* expression resulting in a higher ratio of *scxa* to *sox9a* expression in the tenocytes further away from the cartilage/bone. Balanced co-expression of *scxa* and *sox9a* regulates composition of enthesis ECM. (B) Lack of mechanical force in paralyzed embryos leads to higher levels of *sox9a* and no change in *scxa* expression at the enthesis, leading to a lower ratio of *scxa* to *sox9a* in the tenocytes, which causes changes in the enthesis ECM making it less elastic and more predominantly fibrocartilage.

We also provide evidence that mechanotransduction through TGFβ signaling regulates embryonic entheses. Based on these results, we propose a model for entheseal development in zebrafish in which the transitional nature of enthesis ECM is organized and maintained through an optimal balance of *Scx* and *Sox9* co-expression, which is regulated by mechanical force from muscle contraction through TGFβ signaling in entheseal tenocytes (Fig. 6). Loss of muscle function leads to a loss of force-dependent, TGFβ-mediated repression of *Sox9*, thereby reducing the Scx/Sox9 ratio. Our model agrees with evidence suggesting that *Scx* and *Sox9* are expressed in opposing gradients along the length of more mature entheses in mice, with much higher levels of *Sox9* closer to the bony insertion (Blitz et al., 2013; Kult et al., 2021). It suggests that these patterns of expression arise in embryonic enthesis progenitors and reflect co-expression in individual tenocytes. It further suggests that entheses are specified much earlier than previously reported, with establishment of the graded expression of Sox9a versus Scxa from stiffer to softer tissue domains coinciding with the onset of embryonic muscle contractions. This view also provides a mechanism of feedback between the ECM and the tenocytes that secrete it, as both Scx and Sox9 directly regulate transcription of key ECM components (Bell et al., 1997; Espira et al., 2009; Furumatsu et al., 2010; Leéjard et al., 2007; Subramanian and Schilling, 2015).

Both Scx and Sox9 directly regulate transcription of collagens, proteoglycans and other ECM factors necessary for the organization and function of cartilage and tendon tissues. Whereas Scx drives expression of *Col1a*, Sox9 directly activates expression of *Col2a* (Bell et al., 1997; Espira et al., 2009; Leéjard et al., 2007). Col1a and other components of the tendon ECM enable proper transfer of tensile force to the more fibrous, load bearing Col2a-rich cartilage ECM (Schwartz et al., 2013; Zelzer et al., 2014). Hence, by fine-tuning levels of Scx and Sox9 and their ECM targets, the enthesis tissue is engineered to transition from a soft elastic Col1-rich tissue to a strong fibrous Col2-rich tissue at the cartilage side (Blitz et al., 2013; Sugimoto et al., 2013). Our results demonstrate that in zebrafish this occurs, at least in part, through co-expression of *Scx* and *Sox9* in the same cells, as tendons first become functional in the developing embryo, and that this requires force (Fig. 3). We show that graded assembly of the ECM also involves higher Col1a near the enthesis and tendon mid-body versus higher Tsp4b near the embryonic MTJ. Similarly, Tsp4 expression localizes to tendons and the MTJs of mice (Frolova et al., 2014). Thus our results expand the spectrum of graded ECM components that define different tendon regions (Fig. 1O,P).

Requirements for muscle function in enthesis development in developing limb tendons have been reported in studies of drug-induced paralysis in chick and *Myod1;Myf5* mutant mice that lack muscles (Hall and Herring, 1990; Rot-Nikcevic et al., 2006). However, in both cases defects in long bones and loss of muscles could secondarily disrupt enthesis development. In contrast, paralysis induced by injection of *αBgtx* or mutation of *cacnb1* in zebrafish embryos does not alter the morphologies of the craniofacial cartilage elements or the expression of *sox9a* in chondrocytes, consistent with a direct effect of force on tenocytes (Subramanian et al., 2018; Hoyle et al., 2022). By comparing paralyzed and WT embryos at key stages of zebrafish embryonic craniofacial development, we have identified the stage at which the ratio of *scxa* to *sox9a* responds to force in an enthesis. Coordinated contractions of cranial muscles begin at 60-72 hpf and our data suggest that differences in the ratio of *scxa* and *sox9a* expression between WT and *cacnb1^−/−^* embryos arise due to downregulation of *sox9a* levels in the absence of muscle contraction in *cacnb1^−/−^* embryos. This finding further strengthens our model that force regulates the optimum ratio of *scxa* to *sox9a* in developing entheses by downregulation of *sox9a* expression. Similar to other *in vivo* studies on the roles of muscle contractile force in tendon enthesis development and function (Eliasson et al., 2009; Maeda et al., 2011; Sharir et al., 2011; Shwartz et al., 2012, 2013; Felsenthal and Zelzer, 2017), our studies do not isolate the cells from the surrounding cellular environment and, as such, do not directly measure the effects of force on the cells (Chiquet et al., 2003).

Central to our model is the regulation of TGFβ signaling by force and corresponding changes in the tendon ECM. Similar to our previous studies on trunk tendons, we show that paralysis through injection of *aBgtx* mRNA reduces phosphorylation of SMAD3 in entheseal tenocytes, suggesting that TGFβ signaling at the enthesis in response to mechanical force regulates tenocyte gene expression (Subramanian et al., 2018). Further, we have also shown that stimulation of *aBgtx*-inj embryos rescued the ratio of *scxa* to *sox9a* to WT levels by restoring the repression of *sox9a* expression. Although this stimulation of 2 min is sufficient to rescue the ratio of *scxa* to *sox9a*, it does not appear to be sufficient to change *scxa* expression. Future studies are needed to study the differences in responsivity of *scxa* and *sox9a* transcription to magnitude and duration of muscle activity. We have also shown that force regulates TGFβ signaling in embryonic trunk tenocyte morphogenesis and ECM production (Subramanian et al., 2018). We further show that pharmacological inhibition of TGFβ signaling reduces the ratio of *scxa* to *sox9a* expression at 72 hpf, similar to paralyzed embryos. (Arimura et al., 2017; |Blitz et al., 2013; 2009; Roberts et al., 2019).

Although non-specific and global effects of TGFβ inhibitors are not completely ruled out, we are limited to this approach due to the lack of necessary genetic tools in zebrafish to specifically downregulate or upregulate TGFβ in craniofacial development. We have chosen two different inhibitors and have titrated a dose that does not cause other musculoskeletal defects. Mechanical force activates TGFβ signaling in mammalian tendons and skeletal joints (Arimura et al., 2017; van der Kraan et al., 2012). Lack of joint movement can lead to reduced TGFβ signaling, causing upregulation of proteolytic enzymes in the joint and an increased risk of osteoarthritis. TGFβ can be released from latent TGFβ-binding proteins (LTBPs) in the ECM in response to force (Maeda et al., 2011). Our results are consistent with previous studies of entheses in mice and rats, where both TGFβ signaling and mechanical force regulate the ratio of *Scx* to *Sox9* expression, likely altering ECM composition (Blitz et al., 2013; Saito et al., 2021). However, our results go beyond these studies by investigating transcriptional dynamics at the level of individual cells of the enthesis to show: (1) influences of force on TGFβ signaling in cranial tendons at embryonic stages when entheses first form; (2) that the altered ratios primarily reflect changes in *Sox9* expression; and (3) that the effect of force on entheseal expression of *sox9a* is consistent across different types of entheses. These expression dynamics could reflect interactions between Scx and Sox9 themselves or the ECM proteins they regulate and feedback through TGFβ and other ECM-associated signals. *In vitro* studies have hinted towards a role for Scx in controlling *Sox9* expression in association with E-box transcription factors (Furumatsu et al., 2010). Future development of transgenic lines expressing components of TGFβ signaling is needed to gain better insights into the role of mechanical force on tendon and enthesis development.

In addition to TGFβ, several other signaling pathways have been implicated in enthesis development in mammals, including BMP, FGF, Notch and Shh (Arimura et al., 2017; Blitz et al., 2013; 2009; Roberts et al., 2019). At craniofacial osteo-tendinous entheses, FGF and Notch signaling control the balance of *Scx* and *Sox9* expression, thereby influencing enthesis ECM composition and organization (Roberts et al., 2019). Mechanical force can impact these developmental regulatory signals in tendons. For example, mechanical force from muscle activity regulates the distribution of primary cilia on tenocytes in embryonic and adult entheses that regulate Shh signaling (Fang et al., 2020). In addition to these pathways, mechanotransduction in tendon and cartilage development could be activated by different types and magnitude of forces.

Recent work has shown that the adult enthesis is populated by Gli1^+^ cells, which appear to replace the Scx/Sox9-expressing embryonic enthesis cells (Fang et al., 2020; Felsenthal et al., 2018). This raises several questions relating to the transition from embryonic to adult enthesis development and maintenance. What is the role of the Scx/Sox9-expressing enthesis tenocytes? Perhaps they function at earlier stages to fine-tune the tendon ECM to match the force required for each muscle attachment and thereby establish an ECM template for tendon maturation. If so, what are the cues responsible for their replacement by Gli1^+^ adult enthesis cells? Does TGFβ signaling and the embryonic tendon ECM aid in establishment of the adult enthesis? Future studies, such as spatial transcriptomics to resolve cellular heterogeneity as tendons mature, could provide answers to these fascinating questions.

## MATERIALS AND METHODS

### Zebrafish transgenics and mutants

TgBAC(*scx:mCherry*)*^fb301^*, Tg(*-7.2sox10:gfp*)*^ir937^* transgenic and *cacnb1^−/−ir1092^*;TgBAC(*scxa:mCherry*)*^fb301^* lines have been described previously (Hoffman et al., 2007; Subramanian et al., 2018). All embryos were raised in embryo medium (EM) at 28.5°C and staged as described previously (Kimmel et al., 1995; Westerfield, 2007). Craniofacial muscles and cartilages were labeled as described previously (Schilling and Kimmel, 1997). Adult fish and embryos were collected and processed in accordance with approved University of California, Irvine Institutional Animal Care and Use Committee guidelines.

### mRNA injections and drug treatments

Full-length *aBgtx* mRNA was synthesized from *Pmtb-t7-alpha-bungarotoxin* (*aBgtx*) vector (Megason Lab, Addgene plasmid #69542) following a previously published protocol and injected into Tg(*scx:mCherry*) embryos at the one- to two-cell stage (Swinburne et al., 2015). Stock solution of 10 mM SB431542 (Selleckchem S1067, CID: 4521392) and SB525334 (Selleckchem S1476, CID: 9967941), selective inhibitors of the TGFβ type I (ALK5) receptor were prepared in DMSO and used to obtain a final working concentration of 50 µM in EM. Embryos were incubated in 50 µM SB431542, 50 µM SB525334 or equivalent volume of DMSO for 12 h. Treated embryos were rinsed in pre-warmed (28.5°C) EM before fixation for immunostaining or isHCR protocol.

### Muscle stimulation

Electrical stimulation was used to induce muscle contraction, as previously described (Subramanian et al., 2018; Subramanian and Schilling, 2014). Both *aBgtx*-inj and control embryos or larvae were anesthetized with Tricaine (ethyl 3-aminobenzoate methanesulfonate, Sigma-Aldrich, A5040, SID: 329770864), placed on a silicone plate in EM and stimulated for 2 min at 20 V, 6 msec duration, 4 pulses/sec frequency and 6 msec delay between successive pulses. Stimulated embryos were allowed to recover in EM for 12 h and further processed for immunostaining or isHCR protocol.

### Whole embryo immunohistochemistry

All embryos used for immunofluorescence experiments were fixed in 4% neutral pH buffered paraformaldehyde (PFA, Thermo Fisher Scientific, T353-500, CID: 712) for 2 h at room temperature (25°C) or overnight at 4°C. The embryos were washed with 1× Phosphate Buffered Saline (PBS, CID: 24978514) and permeabilized with cold acetone (Thermo Fisher Scientific, A94, CID: 349996362) for 15 min at −20°C. Following permeabilization, they were rehydrated in PBDT (1×PBS with 2% DMSO, CID 679, Alfa Aesar, 42780) and 1% Triton X-100 (Sigma-Aldrich, T9284, CID: 5590) and processed according to standard antibody staining protocol. Primary antibodies used: rat monoclonal anti-mCherry at 1:500 (Molecular Probes, M11217, RRID: AB_2536611), rabbit anti-pSMAD3 at 1:500 (Antibodies-online, ABIN1043888, RRID: AB_2725792), mouse monoclonal anti-myosin heavy chain (MHC) at 1:250 [Developmental Studies Hybridoma Bank (DSHB), A4.1025], mouse monoclonal anti-Collagen type I (Col1a) at 1:200 (DSHB, 8-3A5, RRID: AB_2877070), mouse monoclonal anti-chicken Collagen type II (Col2a) at 1:200 (DSHB, II-II6B3, RRID: AB_528165) and rabbit anti-Tsp4b at 1:1000 (Subramanian and Schilling, 2014; Webster et al., 1988; De Blas et al., 1984; Linsenmayer and Hendrix, 1980; RRID: AB_2725793). DAPI (Invitrogen, D1306, RRID: AB_2629482) was used at 1:1000 to mark cell nuclei (Subramanian et al., 2018). Preabsorbed secondary antibodies were all obtained from Jackson ImmunoResearch and used for indirect immunofluorescence at 1:1000: Alexa Fluor 488 conjugated donkey anti-rabbit IgG (711-545-152, RRID: 2313584) and Alexa Fluor 594 conjugated donkey anti-rat IgG (712-586-153, RRID: AB_2340691). After staining, embryos were mounted in 1% low melt agarose (CID: 168241) in 1×PBS and imaged.

### *In situ* hybridization chain reaction

Embryos were fixed in 4% neutral pH buffered PFA for 2 h at room temperature (25°C) and used for isHCR protocol as previously published (Trivedi et al., 2018). *Scxa*, *sox9a* and *col1a1a* probes used in this study were custom designed and synthesized by Molecular Instruments, Inc. Following discussion with the technical support team, we increased the concentration of the *scxa* probe set to 3× to improve consistency in signal. isHCR hairpin amplifiers used in this assay were: B1-Alexafluor 488, B2-Alexafluor 546 and B3-Alexafluor 647 (Molecular Instruments Inc.). Following signal development, embryos were mounted in 1% low melt agarose in 5× SSC and imaged.

### Microscopy and image analysis

Embryos processed for fluorescent immunohistochemistry were imaged using a Nikon A1 confocal system (RRID: SCR_020318) with a Nikon Eclipse Ti inverted microscope using a CFI Plan Apochromat VC 60XC (water immersion) objective. Embryos processed for isHCR were imaged using Leica TCS SP8 confocal system (RRID: SCR_018169) with a Leica DMi8 inverted microscope using a Plan Apochromat HC 40× (water immersion) objective. Confocal stacks were collected with system optimized spacing of 0.45 microns and were analyzed using ImageJ (RRID: SCR_003070) and Imaris 10 (RRID: SCR_007370) softwares. Tenocyte progenitors at prospective entheses were identified by their proximity to cartilage chondrocytes. To quantify expression of either *scxa* or *sox9a* in the selected cell, using ImageJ a projection of a *z*-stack comprising the entire cell was created (∼8-10 slices) using DAPI signal to identify the edges of the cell. Quantification of fluorescence in the selected cells was performed by creating ROIs around the DAPI signal corresponding to the tenocyte as previously described (Subramanian et al., 2018). In experiments where Imaris 10 was used, the ROI was created using DAPI as a reference for each tenocyte at each *z*-section. These ROI surfaces were combined and the mean intensity of *scxa*, *sox9a* and *col1a* was measured in these cellular volumes.

### Statistical analysis

Sample size and number of data points required for each experiment were determined using a power analysis calculator (www.powerandsamplesize.com). The embryos were collected from a single tank of fish and processed for injection and downstream stimulation together to minimize variation introduced during handling. Fixation and staining of embryos were also performed together for all samples in a given experiment. Imaging of embryos within each experiment was performed with identical parameters on the same coverslip in a single imaging session. To account for variation between measurements from individual embryos in each sample, we performed linear mixed effects modeling followed by Tukey post hoc pair wise using lmer and lmtest packages in R (Oberpriller et al., 2022). Graphical plots were created in SPSS, Excel and R (RRID: SCR_001905) (using ggplot2 package). Fluorescence intensity to quantify Smad3 phosphorylation was measured as previously described (Subramanian et al., 2018). Measurement of fluorescence intensity was performed for 8-10 cells per embryo (4-6 embryos per condition).

## Supplementary Material

10.1242/develop.201141_sup1 Supplementary information

## References

[DEV201141C1] Akiyama, H., Chaboissier, M.-C., Martin, J. F., Schedl, A. and De Crombrugghe, B. (2002). The transcription factor Sox9 has essential roles in successive steps of the chondrocyte differentiation pathway and is required for expression of *Sox5* and *Sox6*. *Genes Dev.* 16, 2813-2828. 10.1101/gad.1017802 12414734 PMC187468

[DEV201141C2] Akiyama, H., Kim, J.-E., Nakashima, K., Balmes, G., Iwai, N., Deng, J. M., Zhang, Z., Martin, J. F., Behringer, R. R., Nakamura, T. et al. (2005). Osteo-chondroprogenitor cells are derived from Sox9 expressing precursors. *Proc. Natl. Acad. Sci. USA* 102, 14665-14670. 10.1073/pnas.0504750102 16203988 PMC1239942

[DEV201141C3] Apostolakos, J., Durant, T. J. S., Dwyer, C. R., Russel, R. P., Weinreb, J. H., Alaee, F., Beitzel, K., Mccarthy, M. B., Cote, M. P. and Mazzocca, A. D. (2014). The enthesis: a review of the tendon-to-bone insertion. *Muscles Ligaments Tendons J.* 4, 333-342. 10.32098/mltj.03.2014.12 25489552 PMC4241425

[DEV201141C4] Arimura, H., Shukunami, C., Tokunaga, T., Karasugi, T., Okamoto, N., Taniwaki, T., Sakamoto, H., Mizuta, H. and Hiraki, Y. (2017). TGF-β1 improves biomechanical strength by extracellular matrix accumulation without increasing the number of tenogenic lineage cells in a rat rotator cuff repair model. *Am. J. Sports Med.* 45, 2394-2404. 10.1177/0363546517707940 28586631

[DEV201141C5] Bell, D. M., Leung, K. K. H., Wheatley, S. C., Ng, L. J., Zhou, S., Wing Ling, K., Har Sham, M., Koopman, P., Tam, P. P. L. and Cheah, K. S. E. (1997). SOX9 directly regulates the type-II collagen gene. *Nat. Genet.* 16, 174-178. 10.1038/ng0697-174 9171829

[DEV201141C6] Benjamin, M. and Ralphs, J. R. (1998). Fibrocartilage in tendons and ligaments - an adaptation to compressive load. *J. Anat.* 193, 481-494. 10.1046/j.1469-7580.1998.19340481.x 10029181 PMC1467873

[DEV201141C7] Benjamin, M., Kumai, T., Milz, S., Boszczyk, B. M., Boszczyk, A. A. and Ralphs, J. R. (2002). The skeletal attachment of tendons-tendon ‘entheses’. *Comp. Biochem. Physiol. A: Mol. Integr. Physiol.* 133, 931-945. 10.1016/S1095-6433(02)00138-1 12485684

[DEV201141C8] Blitz, E., Viukov, S., Sharir, A., Shwartz, Y., Galloway, J. L., Pryce, B. A., Johnson, R. L., Tabin, C. J., Schweitzer, R. and Zelzer, E. (2009). Bone Ridge Patterning during Musculoskeletal Assembly Is Mediated through SCX Regulation of Bmp4 at the Tendon-Skeleton Junction. *Dev. Cell* 17, 861-873. 10.1016/j.devcel.2009.10.010 20059955 PMC3164485

[DEV201141C9] Blitz, E., Sharir, A., Akiyama, H. and Zelzer, E. (2013). Tendon-bone attachment unit is formed modularly by a distinct pool of *Scx* - and *Sox9* -positive progenitors. *Development* 140, 2680-2690. 10.1242/dev.093906 23720048

[DEV201141C10] Chiquet, M., Renedo, A. S., Huber, F. and Fluck, M. (2003). How do fibroblasts translate mechanical signals into changes in extracellular matrix production? *Matrix Biol.* 22, 73-80. 10.1016/S0945-053X(03)00004-0 12714044

[DEV201141C11] Choi, H. M. T., Calvert, C. R., Husain, N., Huss, D., Barsi, J. C., Deverman, B. E., Hunter, R. C., Kato, M., Lee, S. M., Abelin, A. C. T. et al. (2016). Mapping a multiplexed zoo of mRNA expression. *Development* 143, 3632-3637. 10.1242/dev.140137 27702788 PMC5087610

[DEV201141C12] Cury, D. P., Dia's, F. J., Miglino, M. A. and Watanabe, I. (2016). Structural and ultrastructural characteristics of bone-tendon junction of the calcaneal tendon of adult and elderly wistar rats. *PLoS One* 11, e0153568. 10.1371/journal.pone.0153568 27078690 PMC4831835

[DEV201141C56] De blas, A. (1984). Monoclonal antibodies to specific astroglial and neuronal antigens reveal the cytoarchitecture of the Bergmann glia fibers in the cerebellum. *J. Neurosci.* 4, 265-273. 10.1523/JNEUROSCI.04-01-00265 6693942 PMC6564744

[DEV201141C13] Eliasson, P., Andersson, T. and Aspenburg, P. (2009). Rat Achilles tendon healing: mechanical loading and gene expression. *J. Appl. Physiol.* 107, 399-407. 10.1152/japplphysiol.91563.2008 19541731

[DEV201141C14] Espira, L., Lamoureux, L., Jones, S. C., Gerard, R. D., Dixon, I. M. C. and Czubryt, M. P. (2009). The basic helix–loop–helix transcription factor scleraxis regulates fibroblast collagen synthesis. *J. Mol. Cell. Cardiol.* 47, 188-195. 10.1016/j.yjmcc.2009.03.024 19362560

[DEV201141C15] Fang, F., Schwartz, A. G., Moore, E. R., Sup, M. E. and Thomopoulos, S. (2020). Primary cilia as the nexus of biophysical and hedgehog signaling at the tendon enthesis. *Sci. Adv.* 6, eabc1799. 10.1126/sciadv.abc1799 33127677 PMC7608799

[DEV201141C16] Felsenthal, N. and Zelzer, E. (2017). Mechanical regulation of musculoskeletal system development. *Development* 144, 4271-4283. 10.1242/dev.151266 29183940 PMC6514418

[DEV201141C17] Felsenthal, N., Rubin, S., Stern, T., Krief, S., Pal, D., Pryce, B. A., Schweitzer, R. and Zelzer, E. (2018). Development of migrating tendon-bone attachments involves replacement of progenitor populations. *Development* 145, dev165381. 10.1242/dev.165381 30504126 PMC6307891

[DEV201141C57] Frolova, E. G., Drazba, J., Krukovets, I., Kostenko, V., Blech, L., Harry, C., Vasanji, A., Drumm, C., Sul, P., Jenniskens, G. J., Plow, E. F. and Stenina-Adognravi, O. (2014). Control of organization and function of muscle and tendon by thrombospondin-4. *Matrix Biol.* 37, 35-48. 10.1016/j.matbio.2014.02.003 24589453 PMC4150858

[DEV201141C18] Furumatsu, T., Shukunami, C., Amemiya-Kudo, M., Shimano, H. and Ozaki, T. (2010). Scleraxis and E47 cooperatively regulate the Sox9-dependent transcription. *Int. J. Biochem. Cell Biol.* 42, 148-156. 10.1016/j.biocel.2009.10.003 19828133

[DEV201141C19] Hall, B. K. and Herring, S. W. (1990). Paralysis and growth of the musculoskeletal system in the embryonic chick. *J. Morphol.* 206, 45-56. 10.1002/jmor.1052060105 2246789

[DEV201141C20] Havis, E., Bonnin, M.-A., De Lima, J. E., Charvet, B., Milet, C. and Duprez, D. (2016). TGFβ and FGF promote tendon progenitor fate and act downstream of muscle contraction to regulate tendon differentiation during chick limb development. *Development* 143, 3839-3851. 10.1242/dev.136242 27624906

[DEV201141C21] Hems, T. and Tillmann, B. (2000). Tendon entheses of the human masticatory muscles. *Anat. Embryol.* 202, 201-208. 10.1007/s004290000107 10994993

[DEV201141C53] Hoffman, T. L., Javier, A. L., Campeau, S. A., Knight, R. D. and Schilling, T. F. (2007). Tfap2 transcription factors in zebrafish neural crest development and ectodermal evolution. Journal of Experimental zoology. part B, *Mol. Dev. Evol.* 308, 679-691. 10.1002/jez.b.21189 17724731

[DEV201141C22] Hosseini, A. and Hogg, D. A. (1991). The effects of paralysis on skeletal development in the chick embryo. I. General effects. *J. Anat.* 177, 159-168. 1769890 PMC1260423

[DEV201141C23] Hoyle, D. J., Dranow, D. B. and Schilling, T. F. (2022). Pthlha and mechanical force control early patterning of growth zones in the zebrafish craniofacial skeleton. *Development* 149, 199826. 10.1242/dev.199826 PMC8917414 34919126

[DEV201141C24] Ideo, K., Tokunaga, T., Shukunami, C., Takimoto, A., Yoshimoto, Y., Yonemitsu, R., Karasugi, T., Mizuta, H., Hiraki, Y. and Miyamoto, T. (2020). Role of Scx+/Sox9+ cells as potential progenitor cells for postnatal supraspinatus enthesis formation and healing after injury in mice. *PLOS One* 15, e0242286. 10.1371/journal.pone.0242286 33259516 PMC7707462

[DEV201141C25] Kimmel, C. B., Ballard, W. W., Kimmel, S. R., Ullmann, B. and Schilling, T. F. (1995). Stages of embryonic development of the zebrafish. *Dev. Dyn.* 203, 253-310. 10.1002/aja.1002030302 8589427

[DEV201141C26] Kult, S., Olender, T., Osterwalder, M., Markman, S., Leshkowitz, D., Krief, S., Blecher-Gonen, R., Ben-Moshe, S., Farack, L., Keren-Shaul, H., et al. (2021). Bi-fated tendon-to-bone attachment cells are regulated by shared enhancers and KLF transcription factors. *eLife* 10, e55361. 10.7554/eLife.55361 33448926 PMC7810463

[DEV201141C27] Leéjard, V., Brideau, G., Blais, F., Salingcarnboriboon, R., Wagner, G., Roehrl, M. H. A., Noda, M., Duprez, D., Houillier, P. and Rossert, J. (2007). Scleraxis and NFATc regulate the expression of the Pro-α1(I) Collagen Gene in Tendon Fibroblasts. *J. Biol. Chem.* 282, 17665-17675. 10.1074/jbc.M610113200 17430895

[DEV201141C54] Linsenmayer, T. F. and Hendrix, M. J. C. (1980). Monoclonal antibodies to connective tissue macromolecules: Type II collagen. *Biochem. Biophys. Res. Commun.* 92, 440-446. 10.1016/0006-291x(80)90352-6 7356475

[DEV201141C28] Liu, H., Zhang, C., Zhu, S., Lu, P., Zhu, T., Gong, X., Zhang, Z., Hu, J., Yin, Z., Heng, B. C., et al. (2015). Mohawk promotes the tenogenesis of mesenchymal stem cells through activation of the TGF b signaling pathway. *Stem Cells* 33, 443-455. 10.1002/stem.1866 25332192

[DEV201141C29] Maeda, T., Sakabe, T., Sunaga, A., Sakai, K., Rivera, A. L., Keene, D. R., Sasaki, T., Stavnezer, E., Iannotti, J., Schweitzer, R. et al. (2011). Conversion of mechanical force into TGF-β-mediated biochemical signals. *Curr. Biol.* 21, 933-941. 10.1016/j.cub.2011.04.007 21600772 PMC3118584

[DEV201141C55] Niu, X., Subramanian, A., Hwang, T. H., Schilling, T. F. and Galloway, J. L. (2020). Tendon cell regeneration is mediated by attachment site-resident progenitors and BMP signaling. *Curr. Biol.* 30, 3277-3292. 10.1016/j.cub.2020.06.016 32649909 PMC7484193

[DEV201141C30] Oberpriller, J., Leite, M. D. and Pichler, M. (2022). Fixed or random? On the reliability of mixed-effects models for a small number of levels in grouping variables. *Ecol. Evol.* 12, e9062. 10.1002/ece3.9062 35898418 PMC9309037

[DEV201141C31] Pryce, B. A., Watson, S. S., Murchison, N. D., Staverosky, J. A., DüNker, N. and Schweitzer, R. (2009). Recruitment and maintenance of tendon progenitors by TGFβ signaling are essential for tendon formation. *Development* 136, 1351-1361. 10.1242/dev.027342 19304887 PMC2687466

[DEV201141C32] Roberts, R. R., Bobzin, L., Teng, C. S., Pal, D., Tuzon, C. T., Schweitzer, R. and Merrill, A. E. (2019). FGF signaling patterns cell fate at the interface between tendon and bone. *Development* 146, dev170241. 10.1242/dev.170241 31320326 PMC6703712

[DEV201141C33] Rot-Nikcevic, I., Reddy, T., Downing, K. J., Belliveau, A. C., Hallgrímsson, B., Hall, B. K. and Kablar, B. (2006). Myf5 −/− :MyoD −/− amyogenic fetuses reveal the importance of early contraction and static loading by striated muscle in mouse skeletogenesis. *Dev. Genes Evol.* 216, 1-9. 10.1007/s00427-005-0024-9 16208536

[DEV201141C34] Rufai, A., Ralph's, J. R. and Benjamin, M. (1995). Structure and histopathology of the insertional region of the human achilles tendon. *J. Orthop. Res.* 13, 585-593. 10.1002/jor.1100130414 7674075

[DEV201141C35] Saito, T., Nakamichi, R., Yoshida, A., Hiranaka, T., Okazaki, Y., Nezu, S., Matsuhashi, M., Shimamura, Y., Furumatsu, T., Nishida, K. et al. (2021). The effect of mechanical stress on enthesis homeostasis in a rat Achilles enthesis organ culture model. *J. Orthop. Res.* 40, 1872-1882. 10.1002/jor.25210 34783068

[DEV201141C36] Schilling, T. F. and Kimmel, C. B. (1997). Musculoskeletal patterning in the pharyngeal segments of the zebrafish embryo. *Development* 124, 2945-2960. 10.1242/dev.124.15.2945 9247337

[DEV201141C37] Schwartz, Y., Farlas, Z., Stern, T., Aszodi, A. and Zelzer, E. (2012). Muscle contraction controls skeletal morphogenesis through regulation of chondrocyte convergent extension. *Dev. Biol.* 370, 154-163. 10.1016/j.ydbio.2012.07.026 22884393

[DEV201141C38] Schwartz, A. G., Lipner, J. H., Pasteris, J. D., Genin, G. M. and Thomopoulos, S. (2013). Muscle loading is necessary for the formation of a functional tendon enthesis. *Bone* 55, 44-51. 10.1016/j.bone.2013.03.010 23542869 PMC3650099

[DEV201141C39] Shaw, H. M. and Benjamin, M. (2007). Structure-function relationships of entheses in relation to mechanical load and exercise. *Scand. J. Med. Sci. Sports* 17, 303-315. 10.1111/j.1600-0838.2007.00689.x 17490450

[DEV201141C40] Sharir, A., Stern, T., Rot, C., Shahar, R. and Zelzer, E. (2011). Muscle force regulates bone shaping for optimal loadbearing capacity during embryogenesis. *Development* 138, 3247-3259. 10.1242/dev.063768 21750035

[DEV201141C41] Subramanian, A. and Schilling, T. F. (2014). Thrombospondin-4 controls matrix assembly during development and repair of myotendinous junctions. *eLife* 2014, e02372. 10.7554/eLife.02372 PMC4096842 24941943

[DEV201141C42] Subramanian, A. and Schilling, T. F. (2015). Tendon development and musculoskeletal assembly: emerging roles for the extracellular matrix. *Development* 142, 4191-4204. 10.1242/dev.114777 26672092 PMC4689213

[DEV201141C43] Subramanian, A., Kanzaki, L. F., Galloway, J. L. and Schilling, T. F. (2018). Mechanical force regulates tendon extracellular matrix organization and tenocyte morphogenesis through TGFβ signaling. *eLife* 7, e38069. 10.7554/eLife.38069 30475205 PMC6345564

[DEV201141C44] Sugimoto, Y., Takimoto, A., Akiyama, H., Kist, R., Scherer, G., Nakamura, T., Hiraki, Y. and Shukunami, C. (2013). *Scx+* / *Sox9+* progenitors contribute to the establishment of the junction between cartilage and tendon/ligament. *Development* 140, 2280-2288. 10.1242/dev.096354 23615282

[DEV201141C45] Swinburne, I. A., Mosaliganti, K. R., Green, A. A. and Megason, S. G. (2015). Improved long-term imaging of embryos with genetically encoded α-bungarotoxin. *PLoS One* 10, e0134005. 10.1371/journal.pone.0134005 26244658 PMC4526548

[DEV201141C46] Tadros, A., Huang, B. and Pathria, M. (2018). Muscle-tendon-enthesis unit. *Semin. Musculoskelet. Radiol.* 22, 263-274. 10.1055/s-0038-1641570 29791955

[DEV201141C47] Tan, G.-K., Pryce, B. A., Stabio, A., Brigande, J. V., Wang, C., Xia, Z., Tufa, S. F., Keene, D. R. and Schweitzer, R. (2020). Tgfβ signaling is critical for maintenance of the tendon cell fate. *eLife* 9, e52695. 10.7554/eLife.52695 31961320 PMC7025861

[DEV201141C48] Thomopoulos, S., Genin, G. M. and Galatz, L. M. (2010). The development and morphogenesis of the tendon-to-bone insertion - what development can teach us about healing -. *J. Musculoskelet. Neuronal. Interact.* 10, 35-45. 20190378 PMC3605736

[DEV201141C49] Trivedi, V., Choi, H. M. T., Fraser, S. E. and Pierce, N. A. (2018). Multidimensional quantitative analysis of mRNA expression within intact vertebrate embryos. *Development* 145, e52695. 10.1242/dev.156869 PMC5825878 29311262

[DEV201141C50] Van Der Kraan, P. M., Goumans, M.-J., Blaney Davidson, E. and ten Dijke, P. (2012). Age-dependent alteration of TGF-β signalling in osteoarthritis. *Cell Tissue Res.* 347, 257-265. 10.1007/s00441-011-1194-6 21638205 PMC3250613

[DEV201141C58] Webster, C., Silberstein, L., Hays, A. P. and Blau, H. M. (1988). Fast muscle fibers are preferentially affected in Duchenne muscular dystrophy. *Cell* 52, 503-513. 10.1016/0092-8674(88)90463-1 3342447

[DEV201141C51] Westerfield, M. (2007). *The Zebrafish Book. A Guide for the Laboratory Use of Zebrafish (Danio rerio)*, 5th ed. Eugene: University of Oregon Press.

[DEV201141C52] Zelzer, E., Blitz, E., Killian, M. L. and Thomopoulos, S. (2014). Tendon-to-bone attachment: from development to maturity. *Birth Defects Res. C: Embryo Today Rev.* 102, 101-112. 10.1002/bdrc.21056 PMC4076491 24677726

