# Supplementary material for: Mechanical force regulates Sox9 expression at the developing enthesis
Source: Development. 2023 Aug 18;150(16):dev201141. doi: 10.1242/dev.201141 (PMC10445799; doi:10.1242/dev.201141)
Supplement: Supplementary information [file develop-150-201141-s1.pdf]

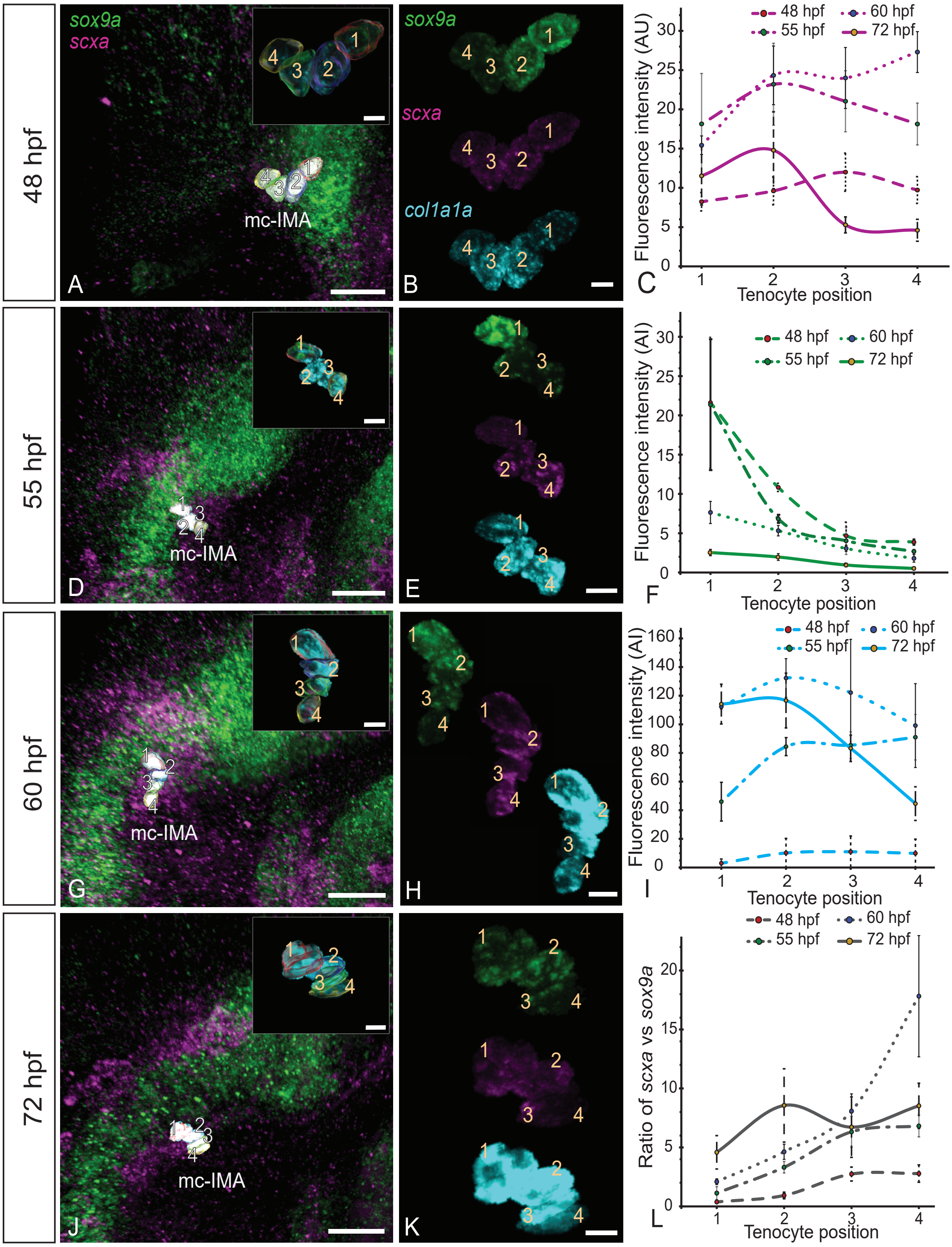


Development • Supplementary information

**Fig. S1. *scxa*, *sox9a* and *col1a1a* expression shows spatial variation in tenocytes at the developing enthesis.** Ventral views, anterior to the left, of the developing Meckel's (mc) cartilages of the lower jaws of zebrafish embryos showing isHCR staining for expression of *scxa* (magenta), *sox9a* (green) and *col1a1a* (cyan) mRNA in tenocytes at specific positions (numbered 1-4, 1 being closest to the cartilage) from the mc-IMA tendon attachment (enthesis) at 48 hpf (A), 55 hpf (D), 60 hpf (G) and 72 hpf (J). Insets show selected cell volume masks determined by DAPI expression that were quantified for *scxa* (C), *sox9a* (F), *col1a1a* (I) expression and used to plot mean fluorescence levels. (B,E,H,K) isHCR showing expression of *scxa*, *sox9a*, and *col1a1a* in each cell volume mask at these developmental stages. (L) Ratios of *scxa* to *sox9a* at each stage were plotted for each tenocyte. N = 4 embryos and 8 cells/embryo at each stage. Error bars show standard deviation. Scale bar (A,D,G,J):20 microns, Scale bar (insets, B,E,H,K): 5 microns.

Development • Supplementary information

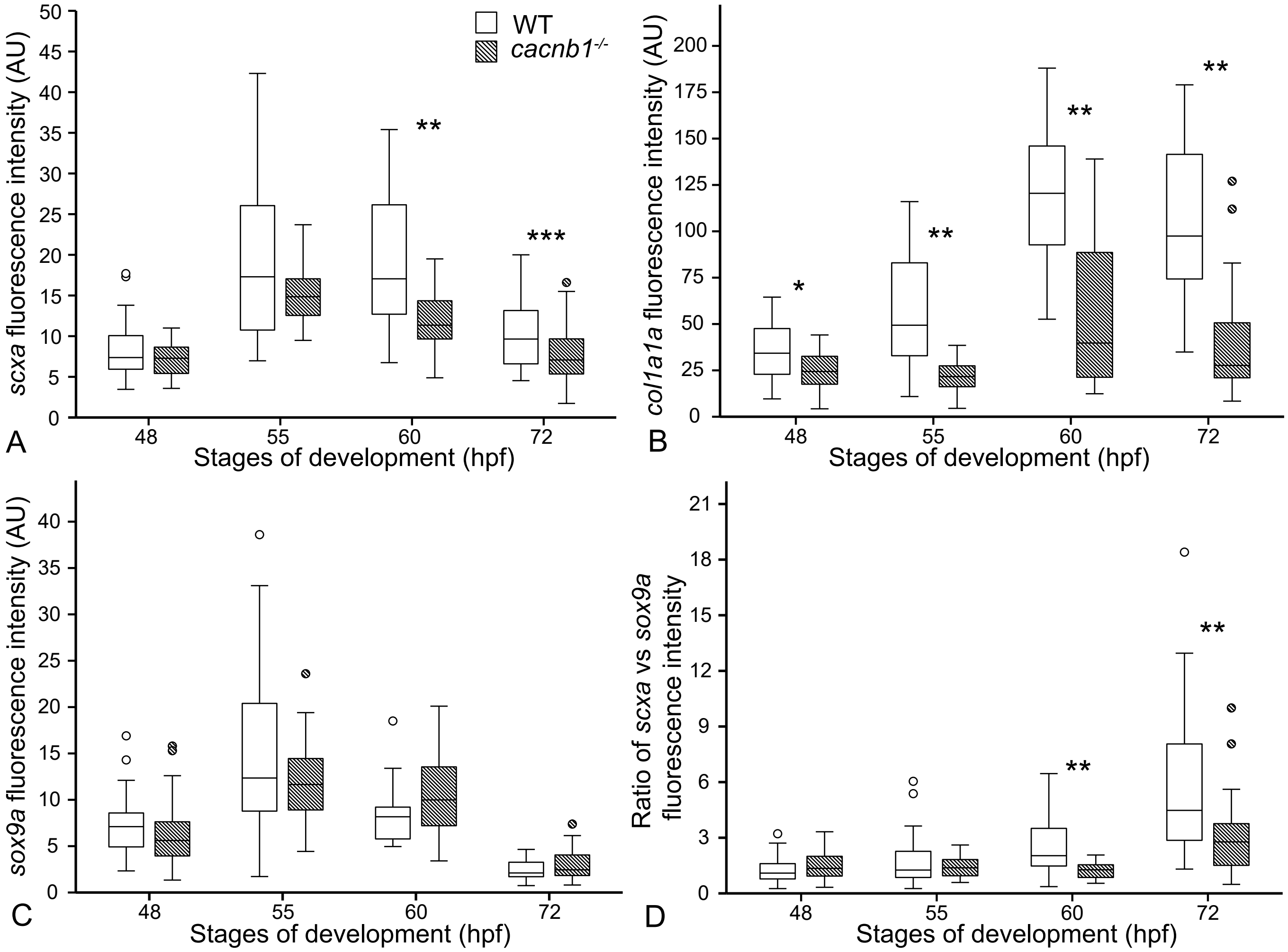


**Fig. S2. Force regulates expression of *scxa*, *sox9a* and *col1a1a* at embryonic cranial enthesis.** Box plots showing quantification of *scxa* (A), *sox9a* (C), *col1a1a* (B) expression and ratio of *scxa* vs *sox9a* expression (D) in WT and *cacnb1*$^{-/-}$ tenocytes. Horizontal line in each box plot shows median value. p-value:*<0.05, **<0.005, ***<0.0005

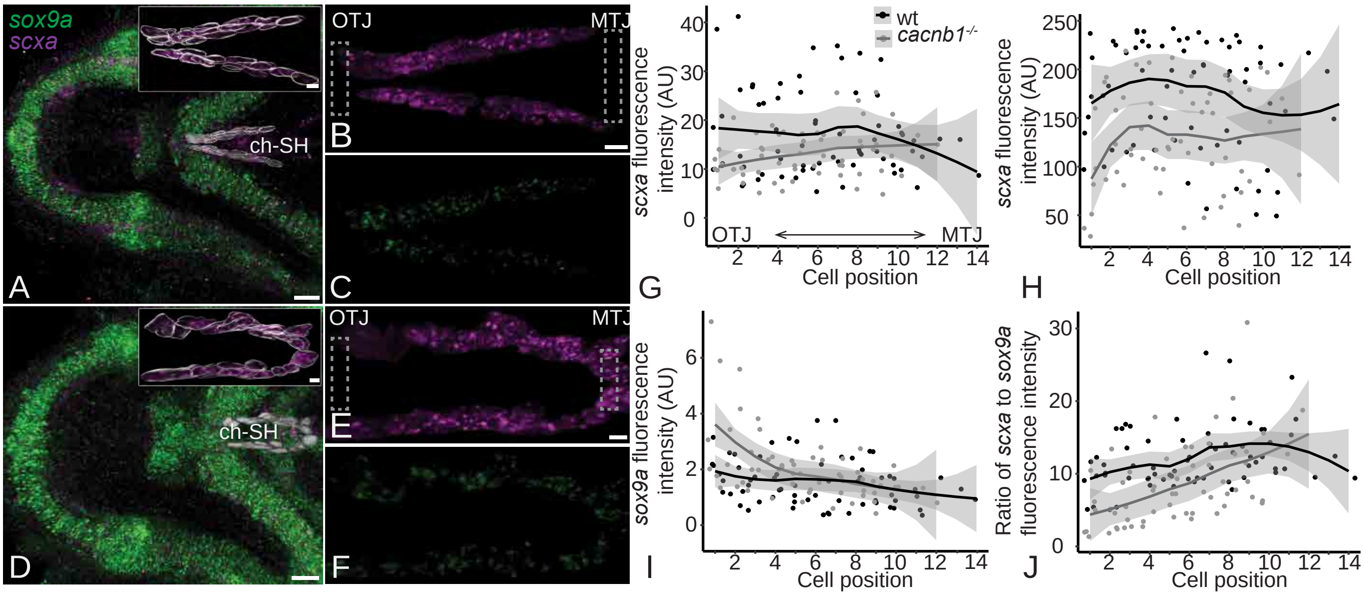


**Fig. S3. Paralysis regulates spatial expression of *scxa*, *sox9a* and *col1a* along the length of the embryonic ch-SH tendon.** (A-F) Ventral views, anterior to the left, of wild type (WT) (A) and *cacnb1*$^{-/-}$ embryos (D) showing isHCR staining for expression of *scxa* (magenta), *sox9a* (green) genes in tenocytes of the ch-SH tendon at 72 hpf. (B,C) Magnified view of WT ch-SH tendon tenocytes showing expression of *scxa* (B) and *sox9a* (C). (E,F) *cacnb1*$^{-/-}$ ch-SH tenocytes expression of *scxa* (E) and *sox9a* (F). Quantification of *scxa* (G), *sox9a* (I), *col1a* (H) expression and ratio of *scxa* vs *sox9a* expression (J) in each selected tenocyte of the ch-SH tendon of WT and *cacnb1*$^{-/-}$ embryo. Regression line was plotted using "loess" method and confidence level is shown around the line in grey. Scale bar (A,D) - 20 microns; (insets, B,C,E,F) - 5 microns.

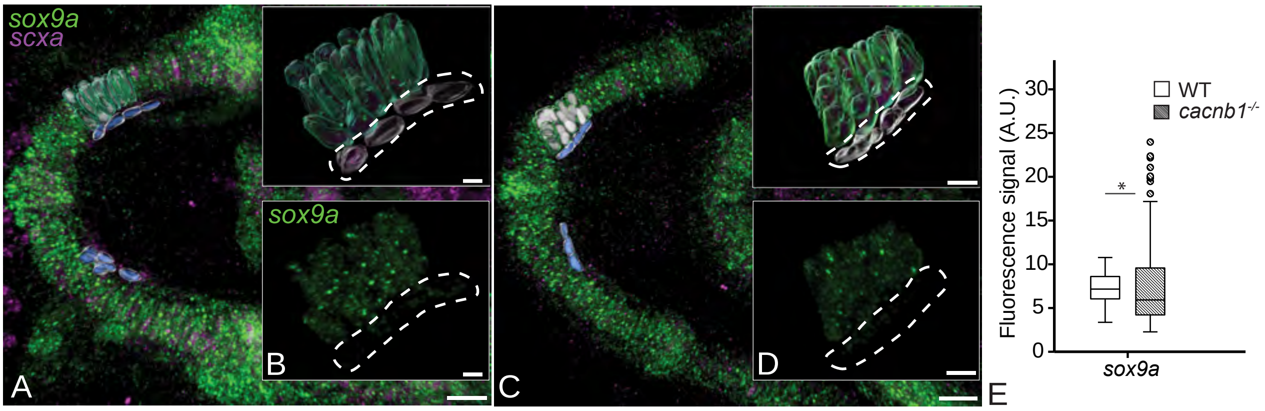


**Fig. S4. Paralysis has a modest effect on *sox9a* expression in chondrocytes at the ch-SH enthesis.** Ventral views, anterior to the left, of the lower jaws of wild type (WT) (A,B) and *cacnb1*$^{-/-}$ embryos (C,D) showing isHCR staining for expression of *scxa* (magenta), *sox9a* (green) genes at tenocytes of the mc-IMA tendon at 72 hpf. DAPI labelled nuclei mark selected chondrocytes (grey) and tenocytes (blue). Dotted line marks the mc-IMA tenocytes in magnified view of WT (B) and *cacnb1*$^{-/-}$ (D). Quantification of *sox9a* (E) expression selected chondrocytes of mc-IMA tendon of WT and *cacnb1*$^{-/-}$ embryos. Horizontal line in each box plot shows median value. N = 3 embryos,15 chondrocytes per embryo. Linear mixed effects model was created and Tukey posthoc pairwise comparison was performed. p value: * - 0.05. Scale bar (A,C) - 20 microns; (insets, B,D) - 5 microns.

Development • Supplementary information

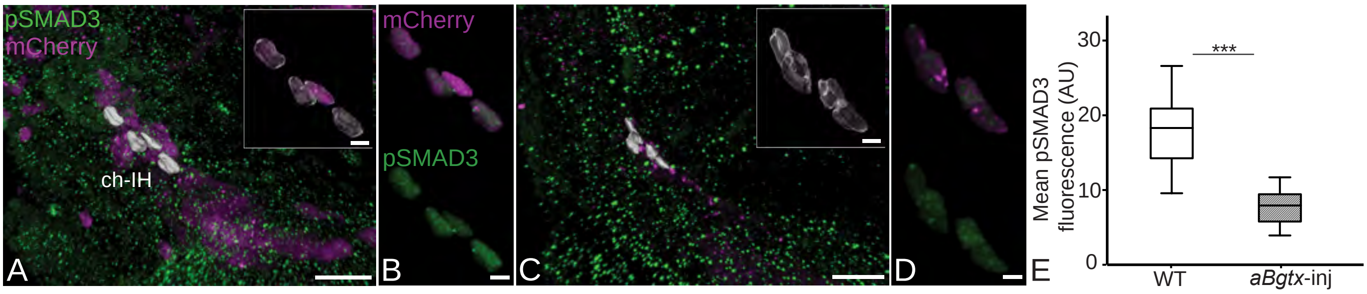


**Fig. S5. Muscle activity regulates SMAD3 phosphorylation in the embryonic enthesis.** (A-D) Ventral views of wild type (WT-NS) (A,B) and *aBgtx*-injected (*aBgtx*-inj) (C,D) embryos at 60 hpf stained for anti-mCherry in *scxa*-expressing tenocytes (magenta) and anti-pSMAD3 (green) at the ch-IH enthesis. (E) Quantification of SMAD3 phosphorylation in the nuclei of entheseal tenocytes of WT and *aBgtx*-inj embryos is shown as a boxplot. Horizontal line in each box plot shows median value. Linear mixed effects model was created and Tukey posthoc pairwise comparison was performed. p-values: *** < 0.001. Scale bar (A,C) - 20 microns; (insets,B,D) - 5 microns.

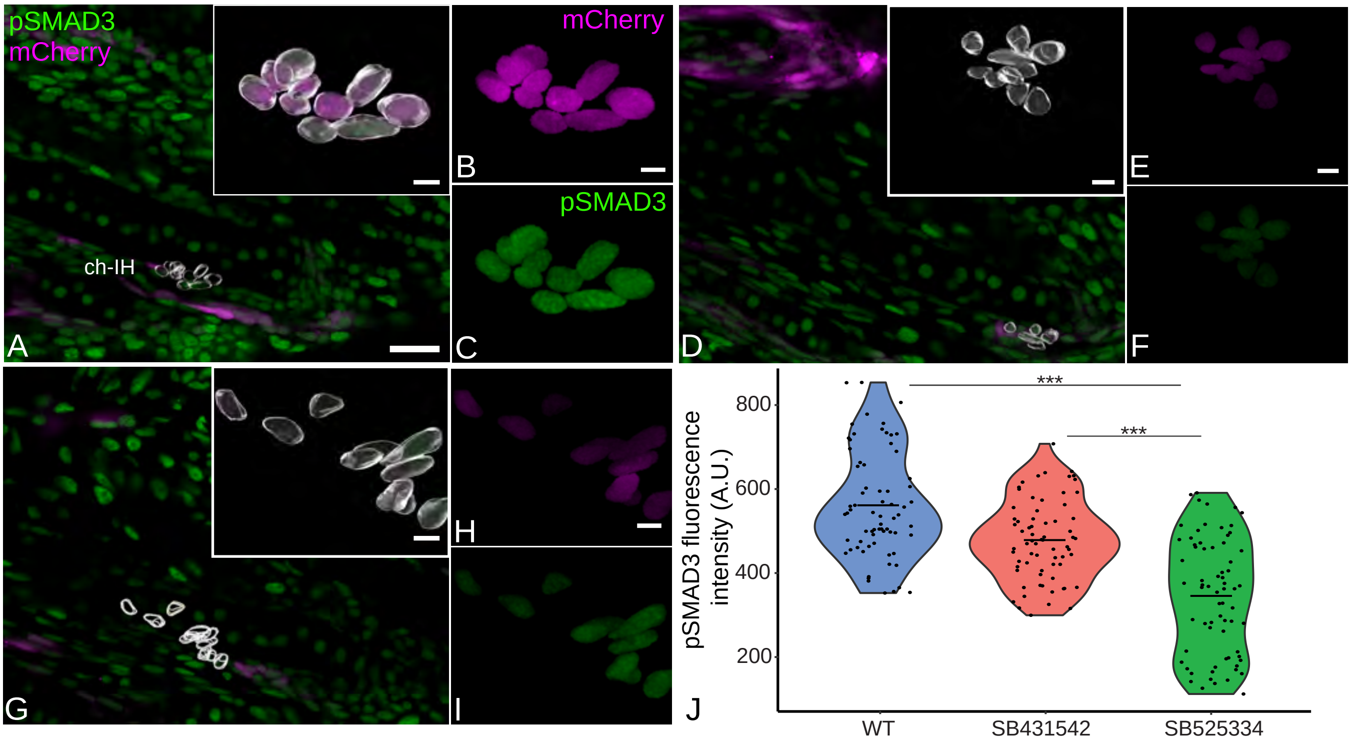


**Fig. S6. TGFbeta inhibition reduces phosphorylation of SMAD3 in entheseal tenocytes.** (A-I) Ventral views, anterior to the left, of 72 hpf-WT (A-C), SB431542-treated (D-F) and SB525334-treated (G-I) zebrafish embryos stained by immunofluorescence for anti-pSMAD3 (A,C,D,F,G,I) and anti-mCherry in *scxa*-expressing tenocytes (A,B,D,E,G,H). Insets show tenocyte nuclear surface volume determined by pSMAD3 immunofluorescence signal. Boxplots depicting fluorescence intensity (A.U.) of pSMAD3 in tenocytes of the ch-IH enthesis in embryos treated with DMSO (WT), SB431542 or SB525334. Horizontal line in each distribution shows median value. N = 4 embryos, 10 cells per embryo. Linear mixed effects model was created and Tukey posthoc pairwise comparison was performed. p-values: *** < 0.001. Scale bar: (A,D,G) 20 microns, (insets, B,C,E,F,H,I) 5 microns.

Development • Supplementary information
